# The Role of Ubiquitination in TWEAK-Stimulated Signaling

**DOI:** 10.3389/fimmu.2013.00472

**Published:** 2013-12-19

**Authors:** Domagoj Vucic

**Affiliations:** ^1^Department of Early Discovery Biochemistry, Genentech, Inc., South San Francisco, CA, USA

**Keywords:** TWEAK, ubiquitin, NF-κB, IAP, c-IAP1, TRAF2, TRAF3, NIK

## Abstract

Tumor necrosis factor superfamily ligands and receptors are responsible for development, immunity, and homeostasis of metazoan organisms. Thus, it is not surprising that signals emanating from these receptors are tightly regulated. Binding of TNF-related weak inducer of apoptosis (TWEAK) to its cognate receptor, FN14, triggers the assembly of receptor-associated signaling complex, which allows the activation of canonical and non-canonical nuclear factor kappa B (NF-κB) as well as mitogen-activated protein kinase signaling pathways. Ubiquitin ligases cellular inhibitor of apoptosis 1 and 2 (c-IAP1 and 2) and adaptor proteins TNFR-associated factors 2 and 3 (TRAF2 and TRAF3) are crucial for the regulation of TWEAK signaling as they facilitate the recruitment of distal signaling components including IKK and linear ubiquitin chain assembly complex complexes. At the same time c-IAP1/2, together with TRAF2 and TRAF3, promote constitutive ubiquitination and proteasomal degradation of NF-κB inducing kinase (NIK) – a kinase with critical role in the activation of non-canonical NF-κB signaling. While c-IAP1/2 mediated ubiquitination allows the activation of TWEAK-stimulated canonical NF-κB signaling, these E3 ligases are negative regulators of non-canonical signaling. TWEAK stimulation prompts the recruitment of c-IAP1/2 as well as TRAF2 and TRAF3 to the FN14 signaling complex leading to c-IAP1/2 autoubiquitination and degradation, which stabilizes NIK and allows subsequent phosphorylation of IKKα and partial proteasomal processing of p100 to activate gene expression. Recent studies have revealed that the spatio-temporal pattern of TWEAK-stimulated ubiquitination is a carefully orchestrated process involving several substrates that are modified by different ubiquitin linkages. Understanding the significance of ubiquitination for TWEAK signaling is important for the overall understanding of TWEAK biology and for the design of therapeutics that can be used in the treatment of human pathologies that are driven by TWEAK/FN14 expression and activity.

## Ubiquitination System

**Figure 1:**
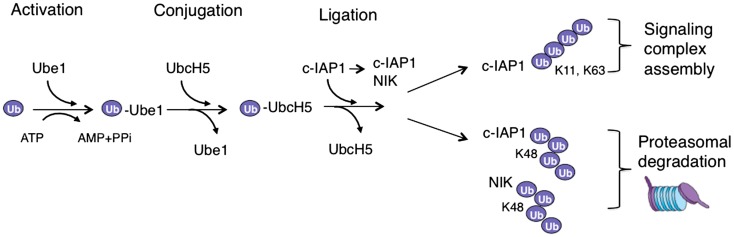
**Ubiquitination cascade in TWEAK signaling**. In the activation reaction ubiquitin is transferred to an E1 enzyme (Ube1) in an ATP-dependent fashion, which leads to the transfer of activated ubiquitin to an E2 enzyme (UbcH5) in the conjugation reaction. The E2 with ubiquitin binds E3 ubiquitin ligase (c-IAP1), which can also bind a substrate – often through a different protein interaction domain – and thus allows the ubiquitin ligation to occur. When polyubiquitin chains are assembled this process is repeated with a lysine (K) residue of the ubiquitin molecule itself serving as a substrate. The assembly of K63- or K11-link polyubiquitin chains on c-IAP1 promotes the formation of signaling complexes, while K48-linked ubiquitination of NIK or c-IAP1 targets them for proteasomal degradation.

The regulated posttranslational modification and degradation of cellular proteins by the ubiquitin-proteasome system impacts a wide range of crucial processes in normal and diseased cells (1). Tumor necrosis factor (TNF) superfamily ligands, including TNF-related weak inducer of apoptosis (TWEAK), rely extensively on ubiquitination to promote activation of non-canonical and canonical nuclear factor kappa B (NF-κB) signaling as well as mitogen-activated protein kinase (MAPK) pathways. Ubiquitination requires the activity of ubiquitin activating enzyme (E1), ubiquitin conjugating enzymes (E2s), and ubiquitin ligases (E3s) (2). Coordinated activity of these components results in the covalent ligation of ubiquitin to the acceptor lysine, or less frequently amino-terminal, residues of the substrate protein (Figure 1). Covalent attachment of a single ubiquitin molecule to the substrate is referred to as monoubiquitination (3). However, the presence of seven lysines and available amino-terminus within a ubiquitin molecule enables the formation of a variety of ubiquitin–ubiquitin linkages and polyubiquitin chains (3). The varied topologies of different polyubiquitin chains provide means for communicating complex biological information that is vital for many cellular functions (4). For example, K63-linked chains, amino-terminally linked chains, and in some cases K11-linked chains, provide a platform for the assembly of signaling complexes (5–7). On the other hand, K48-linked chains mostly target substrate proteins for proteasomal degradation (1).

Hundreds of E3 ligases that ensure substrate specificity and tens of E2 enzymes that dictate the type of the ubiquitin chain assembly present the ubiquitination processes with a remarkable combinatorial potential (8). Numerous ubiquitin-binding domains that recognize different ubiquitin chains and transmit encoded biological information decipher the information embedded in diverse ubiquitin modifications (9). Just like many complex biological systems, ubiquitination is a reversible process. A separate class of enzymes, called deubiquitinases (DUBs), carries out the removal and depolymerization of ubiquitin chains (10). All together, E1–3 enzymes that promote ubiquitination, ubiquitin-binding domains that recognize different ubiquitin moieties, and DUBs that eliminate ubiquitin modifications afford a powerful molecular set of tools for fine-tuning intricate signaling messages.

## Signaling Pathways Induced by TWEAK

Tumor necrosis factor superfamily ligands are homotrimeric type 2 transmembrane proteins that are either membrane-embedded or cleaved to generate soluble proteins (11). These ligands bind one or more members of TNF receptor (TNFR) superfamily, type 1 transmembrane proteins, by interacting with the cysteine-rich domain in the extracellular region of TNFRs (11). Ligands and receptors of TNF/TNFR superfamily are vital for the proper functioning and organization of the immune system (12). Thus, it is no surprise that these proteins are implicated in a variety of genetic or acquired human diseases (12). Binding of TNF ligands to their cognate receptors triggers the assembly of receptor-associated signaling complexes and activates multiple signaling pathways, including the NF-κB, MAPKs c-Jun N-terminal kinase (JNK) and p38, and in some instances cell death (13–15). NF-κB transcription factor family members (NF-κB1 or p105/p50, NF-κB2 or p100/p52, RelA or p65, RelB, and cRel) operate as homodimers or heterodimers and can be activated through the canonical (classical) or non-canonical (alternative) pathways (16). In unstimulated cells inhibitor of canonical NF-κB signaling (IκB) keeps p50/Rela dimer in cytoplasm until IκB kinase β (IKKβ) phosphorylates it marking it for ubiquitination by SCF-βTrCP and subsequent proteasomal degradation (17). Without IκB around NF-κB dimers translocate to the nucleus and stimulate transcription of a series of proinflammatory and anti-apoptotic proteins (17). In the non-canonical pathway NF-κB inducing kinase (NIK) is the primary kinase that phosphorylates IKKα leading to the phosphorylation of the C-terminal domain of NF-κB precursor protein p100 (18). This triggers SCF-βTrCP dependent ubiquitination and partial proteasomal degradation of p100 to yield the mature p52 protein, which together with RelB moves to the nucleus and stimulates the gene expression with largely overlapping pattern with canonical pathway (18).

**Figure 2:**
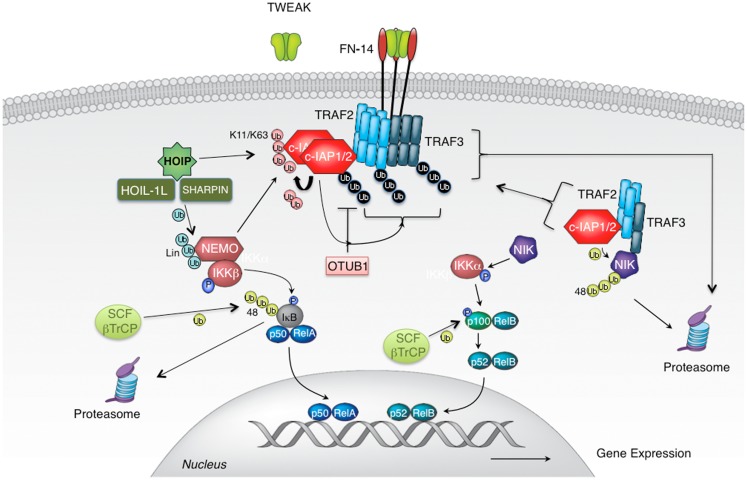
**TWEAK-stimulated activation of canonical and non-canonical NF-κB signaling pathways**. Cellular IAP proteins are positive regulators of canonical and negative regulators of non-canonical NF-κB signaling pathways. c-IAPs, cellular IAPs; FN14, fibroblast growth factor-inducible 14; HOIL-1, heme-oxidized IRP2 ubiquitin ligase-1; HOIP, HOIL-1L-interacting protein/RNF31; Sharpin, SHANK-associated RH domain-interacting protein; IKK, IκB kinase; NEMO, NF-κB essential modulator; NIK, NF-κB inducing kinase; SCF-bTrCP, Skp1/Cul1/F-box β transducin repeat-containing protein; TRAF, tumor necrosis factor (TNF) receptor-associated factor; TWEAK, TNF-related weak inducer of apoptosis; Ub, ubiquitin.

TNF-related weak inducer of apoptosis (also known as TNFSF12) is a TNF family cytokine that binds FN14 receptor (also known as TNFRSF12A) to promote proliferation but also apoptosis in a wide variety of epithelial and endothelial cells (19, 20). Association of TWEAK with FN14 leads to FN14 oligomerization and the recruitment of signaling adaptor proteins TNFR-associated factors 2 and 3 (TRAF2 and TRAF3) (19, 21–23). The presence of TRAF2 in FN14 complex is instrumental for the recruitment of ubiquitin ligases cellular inhibitor of apoptosis 1 and 2 (c-IAP1 and 2), and for the activation of canonical NF-κB and MAPK signaling (24–26) (Figure 2). Adaptor protein TRAF2 exists in cells as a trimer that binds a single molecule of monomeric c-IAP1 or c-IAP2 (27, 28). Engagement of TRAF2 to TWEAK-stimulated signaling complex promotes receptor-mediated TRAF2 aggregation resulting in dimerization/oligomerization of c-IAP proteins thereby upregulating their E3 ligase activity (28, 29). Consequently, c-IAPs ubiquitinate themselves and TRAF2 to enable the recruitment of the IKK complex as well as the linear ubiquitin chain assembly complex (LUBAC) to FN14 (29, 30). The assembly of the TWEAK-FN14-associated receptor complex leads to the rapid activation of canonical NF-κB and MAPKs JNK and p38 signaling pathways within minutes of TWEAK treatment (26, 29) (Figure 2).

Following this first wave of signaling, non-canonical NF-κB signaling gets activated few hours after the formation of TWEAK-FN14 complex (21). The reason for the extended length of time needed for the activation of this pathway lies within the mechanism of activation (18). In the canonical NF-κB all signaling components are poised for action once inhibitory factor IκB has been phosphorylated and degraded. However, in the non-canonical NF-κB pathway, central regulator, kinase NIK, needs to be newly synthesized for the signaling to occur. NIK is kept at extremely low levels in cells by continuous ubiquitination and consequent proteasomal degradation. The protein complex responsible for keeping NIK suppressed consists of adaptor proteins TRAF2 and TRAF3 and E3 ligases c-IAP1 and 2 (31–35). Within this complex TRAF3 at the same time binds NIK and TRAF2 with its constitutive partners c-IAP proteins. This way, TRAF2 and TRAF3 juxtapose E3 ligases c-IAP1/2 and their substrate NIK to enable efficient NIK ubiquitination and suppression of signaling (31) (Figure 2). TWEAK binding to FN14 recruits TRAF2, TRAF3, and with them c-IAP1/2 from the cytoplasm to the membrane-associated receptor complex thereby liberating NIK from degradative control (25, 29, 31). Within hours NIK accumulates and triggers phosphorylation of IKKα, p100, and signaling ensues. However, transient recruitment of TRAF2, TRAF3, and c-IAP proteins at the receptor complex would not permit efficient induction of non-canonical NF-κB signaling. To prevent them from re-entering the cytoplasmic complex with NIK, TRAF2, TRAF3, and c-IAPs need to be sequestered and/or eliminated. For this reason, following receptor engagement these adaptors and E3 ligases relocalize from the cytoplasm into membrane-associated cellular fractions where they undergo ubiquitination in c-IAP E3 ligase dependent manner (25, 29, 36). Consequently, autoubiquitinated c-IAP proteins, as well as ubiquitinated TRAF2 and TRAF3 undergo proteasomal, and in some cases also lysosomal degradation (25, 29). This relocation-ubiquitination-degradation process ensures their efficacious depletion from the cytoplasmic cellular compartment and allows the activation of non-canonical NF-κB signaling.

Similar mechanism for the activation of non-canonical NF-κB signaling is employed by vast majority of TNF family ligands and receptors (for example LT-βR, CD40, CD30) (29). The only exception is B cell-activating factor receptor 3 (BR3 or BAFFR), a receptor for BAFF that exclusively mediates activation of the non-canonical NF-κB pathway (37, 38). Just like the other TNFR family members that activate this signaling pathway, BR3 binds TRAF3 (39, 40). However, BR3 does not bind any other TRAF molecules as other TNFRs do (39, 40). As a result, BR3 does not recruit E3 ligases c-IAP1 and 2 or TRAF6. Being devoid of ubiquitin ligases in its signaling complex, BR3 signaling does not utilize ubiquitination but rather, it relies on the translocation of TRAF3 to the insoluble membrane compartment (29). This sequestration of TRAF3 from the soluble cytoplasmic compartment eliminates the physical link between c-IAPs and NIK and enables activation of the non-canonical NF-κB signaling.

Thus, ubiquitination is instrumental for the proper regulation of the non-canonical NF-κB pathway and TWEAK/FN14 and most of the related TRAF3-binding TNFR family members depend on ubiquitination for effective activation of signaling (29).

## E3 Ligases and Ubiquitin Linkages in TWEAK Signaling

Several ubiquitin ligases have been implicated in TWEAK signaling but probably the most important ones are c-IAP1 and 2 as the elimination or reduction of c-IAP levels severely diminishes canonical NF-κB and MAPK signaling (29). Cellular IAP proteins regulate the activation of canonical NF-κB and MAP kinases following initiation of TWEAK signaling by aggregation and dimerization and induced autoubiquitination within FN14-associated complex (29). Autoubiquitination of c-IAP proteins, and potentially also ubiquitination of adaptor protein TRAF2, provides a platform for the assembly of distal signaling complex that includes NEMO, IKKβ, and HOIL-1L-interacting protein (HOIP) (29). Cellular IAPs promote the assembly of a variety of polyubiquitin linkages on themselves with Lys11-, Lys63-, and Lys48-linked chains being the best studied (41) (Figures 1 and 2). Other polyubiquitin chain linkages as well as branched polyubiquitin chains (involving a mixture of several chain types) possibly also play important function in TWEAK signaling but their role(s) have not been well established yet. Binding of NEMO to these polyubiquitin chains allows the recruitment of IKK complex where activated IKKβ phosphorylates IκBα to stimulate IκBα proteasomal degradation (5, 29). At the same time, association of HOIP with autoubiquitinated c-IAP proteins leads to the engagement of LUBAC complex (29, 42) (Figure 2).

Linear ubiquitin chain assembly complex is an E3 ligase complex that consists of HOIP/RNF31, Heme-oxidized IRP2 ubiquitin ligase-1 (HOIL-1L), and/or SHANK-associated RH domain-interacting protein (Sharpin) (6, 30). It is unique among E3 ligases as it promotes the assembly of linear or Met-linked ubiquitination on NEMO, RIP1, itself, and several other signaling molecules (6). Given that RIP1 does not participate in TWEAK signaling, the activity of LUBAC is likely restricted to NEMO and itself although additional substrates potentially await discovery. Linear ubiquitination stabilizes signaling complexes, and decrease in LUBAC levels, and consequent absence of linear ubiquitination, negatively impacts TWEAK-stimulated canonical NF-κB and MAPK activation (29). Another ubiquitin ligase that has been associated with TWEAK signaling is TRAF6, although not through direct participation in TWEAK-stimulated pathways (43). Starvation of skeletal muscles induces TRAF6 dependent expression of FN14, an event that is critical for the regulation of skeletal muscle atrophy (43).

Non-canonical NF-κB signaling is also critically regulated by ubiquitination and proteasomal degradation. Cytoplasmic complexes consisting of ubiquitin ligases c-IAP1 and 2 and adaptor proteins TRAF2 and TRAF3 promote constitutive ubiquitination and proteasomal degradation of kinase NIK (31–33). Before the enzymatic role of cellular IAP proteins has been demonstrated, it was believed that TRAF2 and TRAF3 possess E3 ligase activity and that TRAF3 is a ubiquitin ligase for NIK (44, 45). However, recent cellular and biochemical studies have indicated that TRAF2 and TRAF3 are not functional E3 ligases and that their RING domains cannot interact with ubiquitin conjugating enzymes or promote ubiquitination (46–48). On the other hand, discoveries that multiple myeloma patients with inactivating mutations in c-IAP1 and 2, as well as knockouts and knockdowns of c-IAP1/2, have constitutive activation of non-canonical NF-κB pathway definitively demonstrated the seminal role of c-IAP E3 ligase activity in the suppression of NIK and non-canonical NF-κB signaling (49–52). In addition, discovery of SMAC-mimicking IAP antagonist compounds that target c-IAP protein for proteasomal degradation greatly aided the affirmation of c-IAPs as E3 ligases for NIK (31, 32). IAP antagonist treatment triggers rapid conformational change in c-IAP proteins that elevates their ubiquitin ligase activity leading to proteasomal degradation (53, 54). Some cells treated with these agents secrete a variety of NF-κB regulated inflammatory cytokines over a prolonged period even in the absence of cell death (55, 56). This cytokine production coincides with processing of p100 to p52 and NIK stabilization thus linking IAP antagonist treatment with the activation of non-canonical NF-κB signaling (55, 56). Presently, several IAP antagonists are undergoing clinical evaluations for anti-cancer treatments and all of them were found to stimulate c-IAP degradation and associated activation of non-canonical NF-κB signaling (57). The exact nature of ubiquitin chains assembled on endogenous NIK has never been determined, probably because of the extremely low levels of ubiquitinated NIK in unstimulated cells. Nevertheless, given that c-IAP1 and 2 can efficiently promote Lys48-linked polyubiquitination that is closely associated with proteasomal degradation, NIK is most likely modified with this chain linkage.

Interestingly, the activation of the non-canonical NF-κB also relies on the c-IAP ubiquitin ligase activity. Stimulation of cells with TWEAK, as well as with the related TNF family ligands that trigger recruitment of TRAF2 and TRAF3 – LIGHT, CD30L, or CD40L leads to the aggregation of c-IAP proteins within the receptor-associated membrane fraction (29). There, c-IAP proteins mediate autoubiquitination of themselves as well as ubiquitination of TRAF2 and TRAF3 causing their proteasomal and lysosomal degradation (25, 29) (Figure 2). TWEAK treatment promotes Lys11-, Lys48-, and Lys63-linked polyubiquitin chain assembly on c-IAP1, and TRAF2 and TRAF3 are likely modified in a similar fashion (41). Sequestration and degradation of c-IAP1/2, TRAF2, and TRAF3 ensures that NIK can accumulate to trigger phosphorylation-mediated activation of signaling. Simultaneous elimination of all NIK regulating components is probably excessive since depletion of any one of those components can break this degradation-promoting circle as seen in multiple myeloma patients that harbor mutations in individual components, IAP antagonists that specifically target c-IAP1/2 proteins or BR3 signaling that selectively recruits TRAF3 (29, 31, 32, 38, 49, 50). Nevertheless, concomitant removal of c-IAP1/2 and TRAF2/3 likely provides added guarantees that ensure the liberation of NIK and activation of signaling. An additional consequence of TWEAK mediated elimination of TRAF2 and c-IAP proteins is the diminished activation of TNF or CD40L stimulated canonical NF-κB and MAPK signaling (36, 58). Since TNFR1 and CD40 rely on c-IAPs and TRAF2 for the assembly of signaling complex and the activation of the canonical NF-κB and MAPK pathways, TWEAK can negatively impact the signaling downstream of TNFR1 and CD40 by depleting critical E3 ligases and adaptors (25, 36, 58).

Processing of p100 to p52 is one of the final steps in the activation of non-canonical NF-κB signaling, and it is also regulated by ubiquitination. IKKα mediated phosphorylation of p100 recruits SCF-βTrCP E3 ligase complex, which promotes p100 ubiquitination and partial proteasomal degradation to yield a p52 form (59–61). In addition, Fbw7, another substrate-binding component of SCF ubiquitin ligase complex, can also regulate proteasomal processing of p100. However, in this case the kinase that provides phosphorylation trigger for ubiquitination is not IKKα but GSK3 (62–65). Thus, multiple kinases and ubiquitin ligases control p100 processing to ensure proper control of the non-canonical NF-κB pathway activation.

## Deubiquitinases in TWEAK Signaling

Given the importance of ubiquitination for TWEAK mediated signaling it is no surprise that deubiquitination also plays a functional role for TWEAK biology. DUBs are enzymes that remove ubiquitin moieties from substrate proteins and allow reversal or inhibition, but in some cases also activation of signaling that is regulated by ubiquitination (7, 10). Recently, the Lys48-specific DUB OTUB1 has been identified as c-IAP1 interacting DUB that can regulate c-IAP1 protein stability following TWEAK stimulation (41, 66). TWEAK stimulates Lys48-linked polyubiquitination of c-IAP1 that ultimately leads to c-IAP1 degradation and the activation of non-canonical NF-κB signaling (31, 41). However, elimination of c-IAP1 also diminishes TWEAK-stimulated activation of canonical NF-κB and MAPK signaling (41). OTUB1 is recruited to TWEAK induced FN14-associated signaling complex where it regulates c-IAP1 Lys48-linked polyubiquitination (41) (Figure 2). In the absence of OTUB1, treatment with TWEAK promotes enhanced c-IAP1 degradation resulting in reduced activation of canonical NF-κB and MAPK pathways (41). However, OTUB1 does not seem to have significant effect on the non-canonical NF-κB pathway, most likely because even in the presence of OTUB1 TWEAK induces c-IAP1 degradation ultimately leading to NIK de-suppression and activation of NF-κB signaling. Another DUB from the ovarian tumor (OTU) domain family of DUBs, A20, potentially regulates non-canonical NF-κB signaling in non-enzymatic fashion by disrupting interaction between c-IAP1 and TRAF2/TRAF3, thereby breaking the link between E3 ligase c-IAP1 and its substrate NIK (67). In the absence of A20, TWEAK-stimulated NIK accumulation and p100 processing were diminished suggesting that A20 is a positive regulator of non-canonical NF-κB signaling (67).

An additional DUB candidate for the regulation of TWEAK-stimulated non-canonical NF-κB signaling is OTUD7B or Cezanne (68). OTUD7B regulates TRAF3 ubiquitination and in particular Lys48-linked polyubiquitination of TRAF3 following stimulation with LT-β or CD40L (69). Through TRAF3 interaction OTUD7B is recruited to CD40 and LT-βR where it regulates TRAF3 ubiquitination and stability. In the absence of OTUD7B TRAF3 is more heavily ubiquitinated with Lys48 linkages leading to its faster degradation (69). As TRAF3 is dispensable for the activation of canonical NF-κB and MAPK signaling OTUD7B does not affect these pathways. However, expedited removal of TRAF3 in the OTUD7B knockouts allows faster activation of non-canonical NF-κB and results in B cell hyper-responsiveness to antigens (69). Although the role of OTUD7B in TWEAK signaling has not been examined yet, striking similarities in the activation of non-canonical NF-κB pathway by TWEAK, LIGHT, and CD40L suggest the OTUD7B might influence TWEAK-stimulated non-canonical NF-κB signaling as well.

## Conclusion

The controlled posttranslational modification of signaling adaptors and effectors has a great potential to regulate signaling outcomes (6, 7, 9, 30). Ubiquitination is one such modification that impacts diverse aspects of TWEAK signaling with direct consequences for the production of inflammatory cytokines and cellular survival and proliferation. TWEAK and FN14 employ signaling principles that significantly rely on ubiquitination for the regulation of signaling complexes and investigation of ubiquitination processes has greatly aided our understanding of the fascinating biology of TWEAK signaling (19). In recent years several agents have been developed that specifically target therapeutically attractive proteins, such as IAP antagonists for E3 ligases c-IAP1/2 and kinase inhibitors for their substrate NIK, in a number of cellular pathways (7, 70). An improved understanding of ubiquitin networks and molecular and physiological mechanisms that control them should reveal novel modalities for targeting TWEAK and FN14 regulated pathways and pathologies.

## Conflict of Interest Statement

The author is an employee of Genentech, Inc.
